# Artificial Intelligence and Machine Learning in Pharmacological Research: Bridging the Gap Between Data and Drug Discovery

**DOI:** 10.7759/cureus.44359

**Published:** 2023-08-30

**Authors:** Shruti Singh, Rajesh Kumar, Shuvasree Payra, Sunil K Singh

**Affiliations:** 1 Department of Pharmacology, All India Institute of Medical Sciences, Patna, IND

**Keywords:** personalized medicine, ai ethics, drug discovery, convoluted neural networks, machine learning, pharmacological research, artificial intelligence

## Abstract

Artificial intelligence (AI) has transformed pharmacological research through machine learning, deep learning, and natural language processing. These advancements have greatly influenced drug discovery, development, and precision medicine. AI algorithms analyze vast biomedical data identifying potential drug targets, predicting efficacy, and optimizing lead compounds. AI has diverse applications in pharmacological research, including target identification, drug repurposing, virtual screening, de novo drug design, toxicity prediction, and personalized medicine. AI improves patient selection, trial design, and real-time data analysis in clinical trials, leading to enhanced safety and efficacy outcomes. Post-marketing surveillance utilizes AI-based systems to monitor adverse events, detect drug interactions, and support pharmacovigilance efforts.

Machine learning models extract patterns from complex datasets, enabling accurate predictions and informed decision-making, thus accelerating drug discovery. Deep learning, specifically convolutional neural networks (CNN), excels in image analysis, aiding biomarker identification and optimizing drug formulation. Natural language processing facilitates the mining and analysis of scientific literature, unlocking valuable insights and information.

However, the adoption of AI in pharmacological research raises ethical considerations. Ensuring data privacy and security, addressing algorithm bias and transparency, obtaining informed consent, and maintaining human oversight in decision-making are crucial ethical concerns. The responsible deployment of AI necessitates robust frameworks and regulations.

The future of AI in pharmacological research is promising, with integration with emerging technologies like genomics, proteomics, and metabolomics offering the potential for personalized medicine and targeted therapies. Collaboration among academia, industry, and regulatory bodies is essential for the ethical implementation of AI in drug discovery and development. Continuous research and development in AI techniques and comprehensive training programs will empower scientists and healthcare professionals to fully exploit AI's potential, leading to improved patient outcomes and innovative pharmacological interventions.

## Introduction and background

Artificial intelligence (AI) is the simulation of human intelligence in machines programmed to think and act like humans. It involves the development of algorithms and computer programs that can perform tasks that typically require human intelligence, such as visual perception, speech recognition, decision-making, and language translation [1]. The field of AI has evolved and expanded, drawing from various academic disciplines such as computer science, mathematics, philosophy, and physics. AI has revolutionized the research landscape across diverse domains, empowering breakthrough discoveries and accelerating progress like never before.

AI techniques, particularly machine learning (ML) and deep learning (DL) have fuelled transformative progress in pharmacological research, overcoming drug development challenges with unmatched momentum, enhanced efficiency, and improved productivity and cost-effectiveness. AI aids in virtual screening, drug design, and drug-target interaction modeling [2]. As a result, AI is applied in various drug discovery stages, including target identification, hit identification, absorption, distribution, metabolism, elimination, toxicity prediction, lead optimization, and drug repositioning [2]. Yet, data availability for robust model training remains a pressing challenge [3]. Ongoing research and advancements aim to overcome challenges and pave the way for a future where AI plays a crucial role in drug discovery and development.

This narrative review aimed to contribute to understanding AI’s role in pharmacology by providing a comprehensive and critical analysis of the existing body of literature and offering insight into its future applications and potential. For a comprehensive analysis of the techniques and tools of artificial intelligence (AI) and their applications, as well as the future prospects and ethical concerns in pharmacological research and precision medicine, a thorough search was performed using appropriate keywords on PubMed, Google Scholar, and ScienceDirect. The objective was to explore the intersection of AI and precision medicine, which promises to revolutionize healthcare by identifying patient-specific treatments and improving outcomes.

## Review

Applications of AI in pharmacology

**Figure 1:**
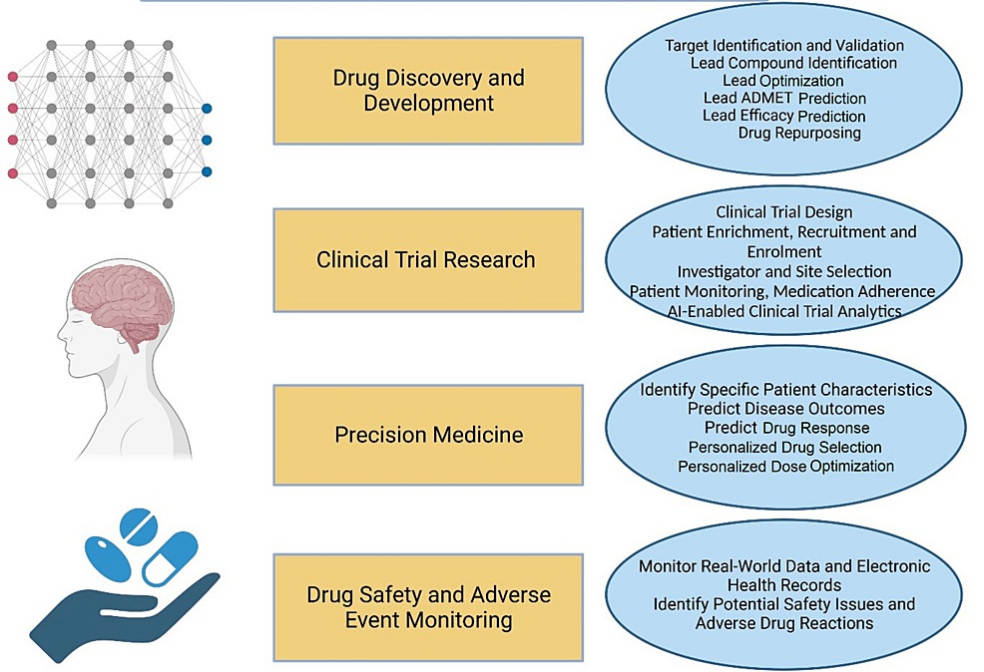
Application of artificial intelligence in pharmacological research and precision medicine. Utilizing machine learning, deep learning (CNN) techniques, and natural language processing AI has revolutionized drug discovery and development (discovery phase, clinical trial phase, and post-marketing surveillance) and precision medicine. CNN: convolutional neural networks The image is created by the authors of this study.

In the application of machine learning, deep learning methods (e.g., convolutional neural network), and natural language processing AI has brought about a groundbreaking transformation in various stages of drug discovery, development (including the discovery phase, clinical trial phase, and post-marketing surveillance) and precision medicine (Figure 1).

Drug discovery and development

Drug discovery and development are crucial in identifying new therapeutic targets, screening potential lead compounds, and assessing drug efficacy and safety. In recent years, AI has emerged as a powerful tool in these areas, revolutionizing the traditional drug discovery process.

AI in Target Identification and Validation

AI revolutionizes target identification and validation in drug discovery, harnessing vast data and computational power. ML and DL algorithms analyze diverse datasets like genomics, proteomics, and clinical data, unveiling promising targets for drug development.

The different approaches used in target identification and validation with the help of AI are as follows: (1) statistical analysis-driven approaches - these employ omics data, including genome-wide association studies (GWAS) and summary data-based Mendelian randomization (SMR), to uncover disease-associated candidate target genes [4]. (2) Network-based approaches - network-based methods reveal intricate biological connections. Gene co-expression and miRNA-disease networks identify disease-associated gene sets and miRNA-disease associations within pathways [5]. Target identification utilizes knowledge graphs, which depict graphs containing entities, relationships, and semantic information, to represent and analyze data [6]. (3) Machine learning-based approaches - ML techniques, including classifiers (e.g., random forest, support vector machine, Neural Net) and regression models, are employed to predict whether a gene is a drug target.

Additionally, AI models can validate potential targets by predicting their druggability and assessing their suitability for therapeutic intervention [7]. This approach reduces the reliance on experimentally validated hypotheses and enables the exploration of previously unexplored targets.

AI-Driven Virtual Screening for Lead Compound Identification

AI revolutionizes virtual screening, expediting compound identification. Models analyze vast chemical databases, predicting compound-target binding likelihood. This prioritizes high-affinity compounds with favorable pharmacokinetic properties efficiently [8-10].

The two main approaches to virtual screening are as follows: (1) structure-based approaches - molecular docking simulations involve a two-step process, conformational space search and scoring. Traditional scoring functions, as well as data-driven machine learning (MLSF) and deep learning-based scoring functions (DLSF), such as 3D convolutional neural networks (3D-CNN) and graph convolutional networks (GCN), prioritize ligand poses and estimate binding affinity. Methods include 3D-voxel-based methods (3D-CNN) for detecting binding pose patterns and molecular graph-based methods (GCN) for model aggregation. (2) Ligand-based approaches assume compounds with similar structures interact with the same target. They employ quantitative structure-activity relationship (QSAR) models, generating molecular descriptors to describe compounds. ML models predict bioactivity using these descriptors. Ligand-based methods include graph-based models (recurrent neural networks {RNNs}, neural graph fingerprints), sequential models (long short-term memory - a type of RNN for sequential compound representation), and similarity-based models (molecular fingerprints, transcriptomic expressions).

In addition to these approaches, chemogenomic methods combine target proteins and compounds to predict drug-target interactions (DTIs). These methods can be similarity-based, focusing on similarities between proteins and compounds, or feature-based, using fixed-length feature vectors to describe targets and compounds. DL models, such as CNNs and deep belief networks, enhance feature-based methods [8-10].

Evotec, a German biotech company, partnered with Exscientia, a UK-based AI-driven drug discovery company. Using Exscientia's "Centaur Chemist" AI design platform, they identified a promising anti-cancer molecule as a drug candidate in just eight months, a fraction of the time traditional methods would require. The AI system analyzed millions of molecules, selecting a few for synthesis, testing, and optimization, leading to the final candidate for clinical trials [11].

AI-Guided Lead Optimization

AI-guided lead optimization offers advantages like reduced human bias, continuous modeling of the chemical space, and overcoming data limitations through transfer learning and semi-supervised learning. These approaches hold promise for generating molecules with desirable properties, potentially accelerating drug design timelines. There are several approaches to AI-guided lead optimization which are mentioned as follows: (1) in the recurrent neural networks (RNN)-based approach, deep generative models learn the chemical space distribution and generate new molecules by mastering simplified molecular input line entry system (SMILES) grammar symbol by symbol. Transfer learning and semi-supervised learning address limited target-specific data. (2) Generative autoencoders like variational autoencoders (VAE) and generative adversarial networks (GAN) are used for molecular design. They learn compressed representations in a latent space, with VAEs introducing a probabilistic element. Conditional VAEs incorporate property vectors for conditional design. Semi-supervised VAEs and prototype-driven diversity networks are other variations used in lead optimization. (3) Reinforcement learning (RL) combines deep generative modeling with RL techniques. It maximizes expected return by formulating molecule generation as an accumulation of rewards. Value learning and policy learning are involved, with the design of reward functions being crucial. RL is utilized in de novo molecular design, and specialized generator architectures like differentiable neural computer (DNC) are used for fine-tuning [12-17].

*AI-Guided Absorption, Distribution, Metabolism, Excretion, and Toxicity (ADMET​*​​​​​​*) Prediction*

AI techniques can effectively predict various drug absorption, distribution, metabolism, excretion, and toxicity properties. Predictive models utilize engineered and learned molecular descriptors to accurately forecast human intestinal absorption (HIA). In vitro assays, such as Caco-2 cell permeability and parallel artificial membrane permeability, predict the potential for absorption. Comprehensive datasets enable the development of predictive models for drug-protein binding, P-gp inhibition, and blood-brain barrier (BBB) permeability. By exploring ML models, molecular descriptors, and structural patterns, precise predictions for BBB permeability, cytochrome P450 (CYP) enzyme substrate and inhibitor interactions, plasma half-life, solubility, metabolic stability, potential metabolites, renal excretion, bile salt export pump (BSEP) inhibition, hepatotoxicity assessment, and cardiotoxicity can be achieved [18-29].

Prediction of Drug Efficacy and Safety Using AI Models

AI models utilize preclinical and clinical studies data to predict various drug properties. Using ML algorithms, they analyze large datasets and identify molecular features associated with therapeutic response and toxicity, helping select promising drug candidates. Drug-drug interactions are a significant concern in drug development, and accurate prediction of cytochrome P450 (CYP450) enzyme inhibition is crucial. In a study, Wu et al. utilized ensemble learning and DL techniques to classify CYP450 inhibitors [30]. Ensemble models, including random forest, gradient boosting decision tree, and eXtreme gradient boosting, outperformed DL, achieving an accuracy of 90.4% [30]. The SHapley Additive exPlanations (SHAP) method was used to interpret the models and identify potential drug-drug interactions during early drug discovery.

AI-Guided Drug Repositioning

AI-guided drug repositioning involves network-based approaches (clustering, network) and DL algorithms, such as deep neural networks (DNN) and convolutional neural networks (CNN), to identify new indications for existing drugs [31-34]. These computational methods access heterogeneous data and patterns in drug-disease associations to prioritize and predict potential drug repositioning candidates. Wu et al. used graph clustering algorithms to identify drug repositioning candidates by constructing a weighted heterogeneous network and considering shared genes for features [35]. Gottlieb et al. constructed a PREDICT classification model using logistic regression and multiple similarity measures to predict drug-disease associations based on ML [36]. Napolitano et al. used gene expression signatures, drug structures, and target proteins to calculate drug similarities and trained a multiclass support vector machines (SVM) model for drug-disease association prediction [37].

Clinical trial research

Protocol Design and Reporting

AI systems extract valuable patterns of information to inform and enhance trial design. The SPIRIT-AI extension provides reporting guidelines for clinical trials evaluating interventions with an AI component [38-40]. These guidelines enhance transparency, consistency, and interpretability by improving protocol reporting and providing evidence-based recommendations for addressing essential elements.

Patient Selection and Recruitment

AI-assisted techniques and digital transformation enable precise patient identification, optimize cohort composition, and enhance recruitment and retention rates in clinical trials. Automation and ML use large datasets, including electronic health records and omics data, to make intelligent predictions and streamline patient selection. This results in improved trial enrollment and retention, ultimately enhancing the efficiency of clinical trials.

Investigator and Site Selection

AI technologies aid in selecting high-functioning investigator sites for clinical trials. They identify target locations, qualified investigators, and priority candidates while ensuring compliance with Good Clinical Practice requirements. AI also helps collect and collate evidence to satisfy regulators regarding study timelines, data quality, and integrity, improving the trial process.

Monitoring and Management of Clinical Trials

AI algorithms analyze real-time patient data, ensuring trial integrity and identifying adverse events. Automation and ML optimize data collection, improve quality, and enable real-time monitoring for higher trial success rates. AI enhances patient monitoring, medication adherence, and retention through data automation, digital assessments, and real-time insights from wearable technology, enhancing engagement and retention.

Image Analysis and Biomarker Computation

AI techniques enable automated and precise medical imaging analysis, facilitating the identification of patterns, abnormalities, and disease-specific biomarkers. Integrating AI with imaging biomarker analysis pipelines improves image-based evaluations' accuracy, consistency, and efficiency in clinical trials.

Intelligent Data Collection and Management

AI streamlines data collection and management in clinical trials, accelerating the process. Collecting data through automated processes and utilizing this for AI algorithms minimizes errors, extracts relevant information from diverse sources, and data becomes efficiently structured and organized. Real-time access to data enhances analysis and decision-making, expediting trials and improving efficiency [40-42].

Pharmacoepidemiology and pharmacovigilance

AI techniques in pharmacovigilance advance drug safety monitoring by analyzing real-world data. Using ML and natural language processing (NLP), AI predicts and detects adverse drug events (ADEs), improving medication-related problem detection from diverse data sources like electronic health records and pharmacovigilance databases. AI also enhances efficiency and consistency in processing individual case safety reports (ICSRs), automating manual processes, removing bias, and providing valuable insights for data scientists and medical professionals. AI-based methods in pharmacovigilance extend beyond data analysis, encompassing adverse event detection, risk assessment, and signal detection during post-marketing surveillance. Automation streamlines adverse event case processing, including extraction from source documents and case validity evaluation, improving the efficiency and quality of pharmacovigilance activities [43-48].

In a study, Fan et al. utilize DL techniques and publicly available data to improve the detection and identification of unreported drug side effects. By applying a DL-based approach using Bidirectional Encoder Representations from Transformers (BERT) models on a dataset of 10,000 reviews from WebMD and Drugs.com, the proposed model achieves state-of-the-art performance in ADE detection and extraction. The study showcases the potential of DL in healthcare tasks and information extraction, providing a solution to the challenges doctors face when prescribing drugs [49]. Personalized medicine, driven by advancements in AI and genomic data integration, has revolutionized healthcare by tailoring treatments to individual patients.

AI Models for Predicting Drug Response and Optimizing Treatment Outcomes

AI models optimize treatment outcomes by predicting drug response using ML algorithms and analyzing diverse biomedical data. They identify molecular signatures and phenotypic changes linked to drug response, enabling personalized therapies and drug repurposing. AI models also uncover biological mechanisms of drug response, leading to the development of new therapeutics and targeted interventions. Their use enhances clinical trial success rates and improves drug efficacy and safety predictions.

Integration of Genomic Data and AI Algorithms for Personalized Drug Selection

Integrating genomic data and AI algorithms enables personalized drug selection by analyzing genetic variants associated with drug response and adverse reactions. This approach enhances personalized medicine by avoiding ineffective or harmful medications for specific patients [50,51].

Individualized patient care is crucial in epilepsy treatment, considering that around 30% of epilepsy patients do not achieve sufficient control with available anti-epileptic drugs (AEDs). This leads to challenges such as comorbid illnesses, reduced quality of life, increased mortality risk, and higher treatment costs. A comprehensive understanding and prediction of AED response are needed to overcome these challenges. Previous research has mainly focused on specific genes related to drug metabolism, neglecting other genetic factors and disease mechanisms. However, considering multiple factors, a holistic approach is required in precision medicine. ML techniques can also integrate clinical and genetic data to predict drug response. In a study on brivaracetam, researchers successfully developed predictive models for drug response by combining ML with clinical and genetic data from 235 patients. This study highlights the potential of integrating high-dimensional genetics data with clinical information to predict AED response and optimize treatment outcomes [52].

AI-Guided Dosage Optimization for Individual Patients

AI-guided dosage optimization is essential for precision pharmacotherapy. Patient-specific characteristics (age, comorbidities) and data, including clinical information and biomarkers, are analyzed by AI algorithms to determine optimal drug dosages. ML techniques help identify patterns and correlations, enabling personalized dosing regimens for better efficacy and safety.

CURATE.AI is an AI-powered platform for personalized medicine that optimizes treatment outcomes by considering individual patient characteristics. It analyzes patient-specific data, including treatment response and changes in condition, to provide personalized dosing recommendations. The platform dynamically adjusts chemotherapy doses for cancer patients, aiming for optimal efficacy and minimal toxicity. CURATE.AI has potential applications in hypertension, diabetes, and digital therapeutics. The platform harnesses AI to provide tailored dosing recommendations, improving patient care and treatment outcomes [53].

AI techniques and methods in pharmacology

Several AI tools and techniques exist that can be classified on the basis of neural networks, use of training data, and extraction of features, e.g., machine learning vs deep learning, supervised vs unsupervised learning, and feature selection vs dimensionality reduction, respectively. Machine learning and deep learning are related but distinct concepts within the artificial intelligence (AI) field (Table 1).

**Table 1:** Artificial intelligence (AI) techniques and uses in pharmacological research. LASSO: Least Absolute Shrinkage and Selection Operation

Technique	Methods	Process
Machine learning	Support vector machine (SVM)	It finds the best hyperplane that separates different classes with the maximum margin, allowing effective classification even in complex datasets. Identifies decision boundaries in high-dimensional data and can handle non-linear relationships. It is used for classification and regression tasks in pharmacological research.
	Random forest	An ensemble learning method, that works by combining multiple decision trees to improve predictive accuracy, feature importance analysis, and identification of potential drug candidates. Primarily used for feature selection and classification tasks in drug discovery and toxicity prediction.
	Supervised learning	It involves training models with labeled data to yield desired outputs, enabling accurate classification and prediction tasks through algorithms, such as neural networks, support vector machines, and random forests. Drug-target interaction prediction, virtual screening, and toxicity prediction are its main uses.
	Unsupervised learning principal component analysis (PCA)	It involves finding patterns and structures in unlabeled data without explicit human supervision. It includes tasks like clustering, association, and dimensionality reduction to discover insights and extract meaningful information from the data. It is utilized for target identification, lead identification and optimization, pharmacovigilance and adverse drug event (ADE) detection, drug repurposing, bioactivity prediction
	Reinforcement learning	Reinforcement learning is the science of decision-making through trial and error, where an agent learns optimal behavior in an environment to maximize reward. It involves an agent exploring and interacting with an environment, learning from outcomes, and adjusting its actions based on feedback. It has great value in personalized medicine and dose optimization.
	Feature selection recursive feature elimination (RFE) LASSO regression	Recursive feature elimination (RFE) is a feature selection algorithm that iteratively eliminates less important features from a dataset based on their relevance in predicting the target variable. It starts with all features and removes them one by one until the desired number of features is reached. LASSO regression, also known as L1 regularization, is a linear regression technique that performs both feature selection and regularization by adding a penalty term to the loss function. It encourages sparsity in the coefficients, effectively shrinking less important features to zero, and keeping only the most relevant features in the model. They are primarily used to select relevant molecular or clinical descriptors for drug-target interaction prediction or patient stratification.
	Dimensionality reduction principal component analysis (PCA) t-SNE (t-distributed stochastic neighbor embedding)	Utilized to transform high-dimensional data into lower-dimensional representations while preserving essential information. It has value in E data exploration and visualization, feature selection, clustering and classification, noise reduction in data, and pre-processing for machine learning.
Deep learning	Neural networks	Mimic the structure and function of biological neurons.
	Convolutional neural networks (CNNs)	They employ convolution, a mathematical operation, to process pixel data. By breaking down images into smaller features and progressively combining them into more complex patterns, CNNs efficiently learn and extract abstract representations, minimizing overfitting. These have revolutionized image analysis tasks, enabling accurate image classification, segmentation, and object detection.
	Recurrent neural networks (RNNs)	Sequence-based tasks like protein structure prediction.

Machine learning refers to a subset of AI techniques where computers learn from data and improve their performance without being explicitly programmed. It involves the development of algorithms and models that can automatically learn and make predictions or decisions based on data.

On the other hand, deep learning is a subfield of machine learning that focuses on training artificial neural networks to mimic the human brain's learning process. It involves using deep neural networks, composed of multiple layers of interconnected artificial neurons, to process and analyze complex patterns in data. Deep learning algorithms can automatically learn hierarchical representations of data and have shown remarkable performance in tasks such as image recognition, natural language processing, and speech recognition.

In summary, machine learning is a broader concept encompassing various algorithms and techniques for training computers to learn from data. In contrast, deep learning is a specific approach within machine learning that focuses on training deep neural networks to learn and extract complex patterns from data.

Machine learning

**Figure 2:**
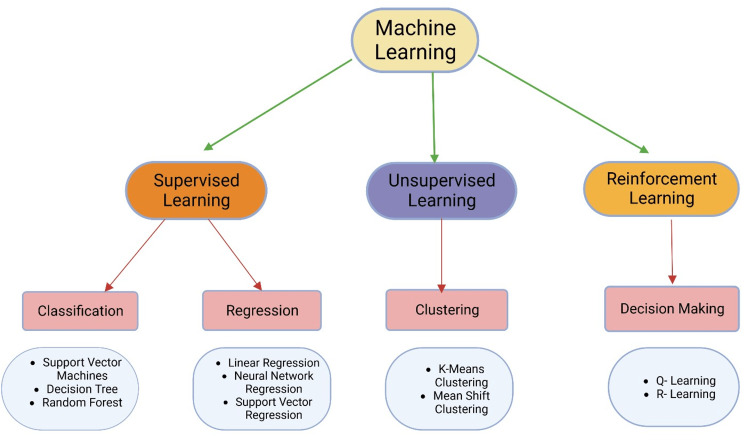
Types of machine learning. Machine learning encompasses three main types - supervised, unsupervised, and reinforcement. Supervised learning involves classification and regression, where models are trained with labeled data. Unsupervised learning focuses on clustering and finding patterns in unlabeled data. Reinforcement learning improves model performance through interaction with the environment. In the provided visualization, colored dots and triangles represent training data, while yellow stars symbolize new data that can be predicted by the trained model. The image is created by the authors of this study.

Machine learning has become increasingly important in pharmacological research, aiding in discovering new drugs, predicting drug responses, and optimizing treatment regimens. ML algorithms analyze large-scale biological and chemical databases, extract meaningful patterns, and make accurate predictions (Table 2) [4]. Several ML types find applications in pharmacology (Figure 2).

**Table 2:** Key databases for pharmacological research. There are several databases that artificial intelligence (AI) leverages to aid in drug discovery and precision medicine. A few are mentioned here. TCGA: The Cancer Genome Atlas; ADMET: absorption, distribution, metabolism, excretion, and toxicity

Database	Description	Used for
LinkedOmics	Comprehensive database of cancer clinical and molecular data. It gathers TCGA cancer-related multi-omics, clinical, and mass spectrometry proteomics data.	Target identification
DepMap portal	Website portal offering analytical and visualization tools for cancer. It includes cancer cell line sensitivity and genetic data.	Target identification
Therapeutic target database	Database of linked medications and recognized therapeutic proteins, nucleic acids, and diseases.	Target identification
DUD-E	Programs for benchmarking molecular docking by providing challenging decoy.	Hit identification
CSAR	Benchmark databases of protein-ligand complexes with various crystal structures and binding affinities.	Hit identification
BindingDB	An online database of measured binding affinities focused primarily on the interaction of drug target proteins with small drug-like molecules.	Hit identification, ADMET property prediction
DrugBank	Free comprehensive database of drugs and drug targets. It contains different chemicals and target information for each drug.	Hit identification, ADMET property prediction, training deep generative models
MATADOR	Integrated medication information on medical indications, adverse drug effects, drug metabolism, target protein pathways, and gene ontology terms.	Hit identification
PubChem	Integrated chemistry database. It includes small to large molecules with structure, physical properties, bioactivity, patents, etc.	Hit identification, ADMET property prediction, training deep generative models
ChemIDplus	An online search portal that provides access to chemicals listed in the National Library of Medicine databases	ADMET property prediction
ToxRefDB	Data were collected from more than 5000 in vivo toxicity studies, to contain 10 types of toxicity studies.	ADMET property prediction
GDB-13	A fully cataloged virtual database based on simple chemical stability and synthetic feasibility, up to 13 atoms C, N, O, S, and Cl.	Training deep generative models
GDB-17	Fully cataloged virtual database based on simple chemical stability and synthetic feasibility, up to 17 C, N, O, S, and halogen atoms	Training deep generative models
ChEMBL	A curated database of bioactive drug-like small molecules. It mainly covers 2D structures, calculated properties, and bioactivity.	Hit identification, ADMET property prediction, training deep generative models

Support Vector Machines (SVM)

**Figure 3:**
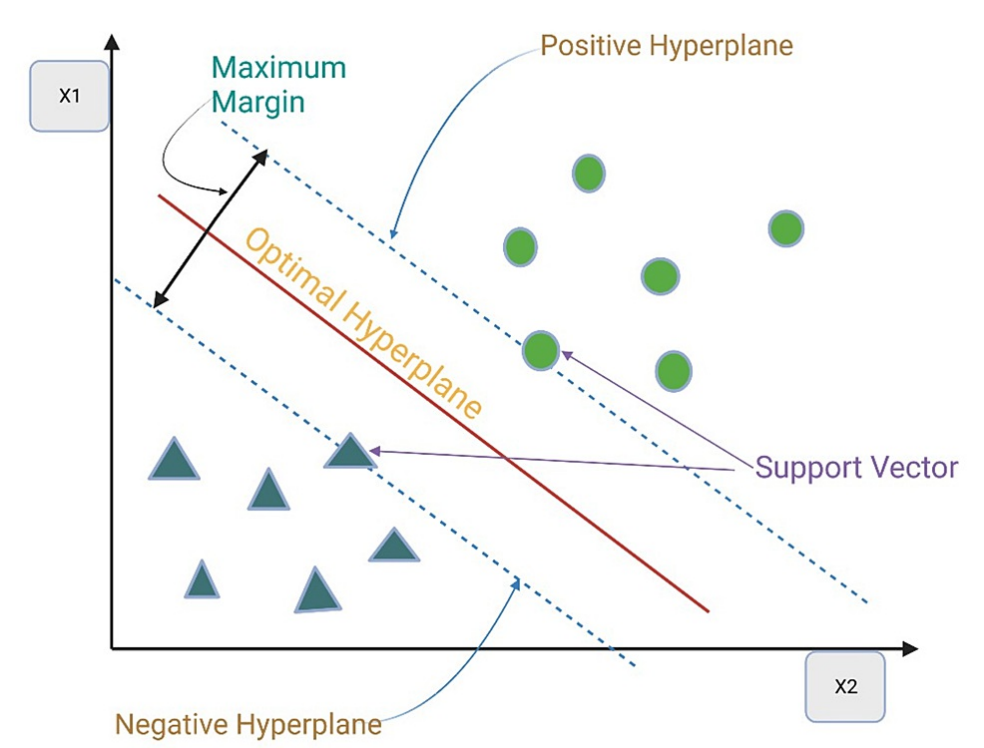
Support vector machine technique in artificial intelligence (AI). Support vector machines (SVM) is a popular supervised learning algorithm for classification and regression problems. It aims to create a hyperplane, which is a decision boundary, to separate data points into different classes in n-dimensional space. The hyperplane is determined by selecting support vectors, which are the closest data points to the boundary. The goal is to find the hyperplane with the maximum margin, or distance, between the classes. The hyperplane’s dimensions depend on the dataset's number of features. Support vectors play a crucial role in determining the position of the hyperplane. They are the data points that support or influence the location of the boundary. The image is created by the authors of this study.

SVM is a robust algorithm for classification and regression tasks in pharmacological research. It identifies decision boundaries in high-dimensional data and can handle non-linear relationships (Table 1 and Figure 3) [54].

In recent research on chemoinformatics and drug discovery, the SVM algorithm was employed to identify potential inhibitors for two important drug targets: thrombin and histone deacetylase 1 (HDAC1). Thrombin is a critical enzyme involved in blood coagulation, while HDAC1 plays a key role in gene regulation and is a promising target for cancer treatment. The SVM algorithm, known for its versatility in predicting molecular properties, was utilized to classify compounds as potential HDAC1 inhibitors. Additionally, a pharmacophore model based on zinc-binding groups (ZBG) was created to aid in identifying HDAC1 inhibitors. The resulting hits from the SVM and pharmacophore models underwent molecular docking analysis to evaluate their binding affinity to HDAC1. Through this screening process, a set of twenty-three compounds was selected and further tested, leading to the discovery of three compounds with HDAC1 inhibition and moderate anti-proliferative activity [55]. These findings represent significant progress in drug discovery, offering potential implications for developing therapeutic agents targeting blood clotting disorders and certain cancers.

Random Forest

**Figure 4:**
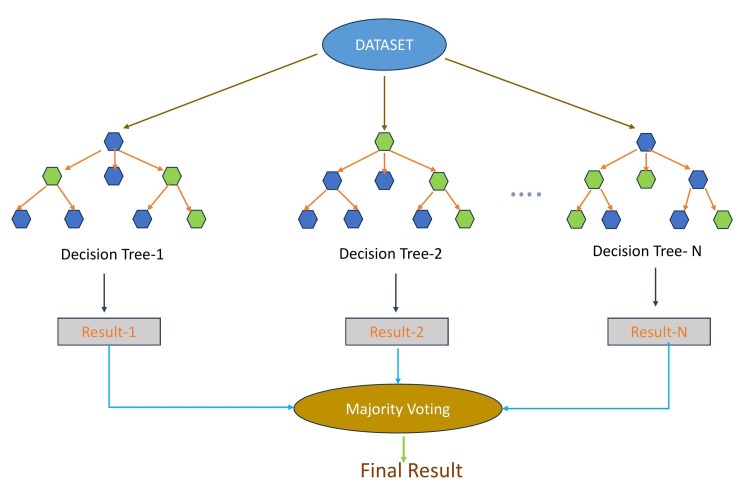
Random forest technique in artificial intelligence (AI). Random forest is a supervised learning algorithm for classification and regression tasks. It utilizes ensemble learning by combining multiple decision trees to enhance predictive accuracy. Each tree provides a prediction, and the final output is determined by majority voting. Increasing the number of trees improves accuracy and prevents overfitting. The image is created by the authors of this study.

Random forest is a popular supervised learning algorithm widely used in pharmacological research. It combines multiple decision trees to make predictions and is known for its robustness and ability to handle high-dimensional data. Random forests have been applied in virtual screening to identify potential drug candidates efficiently. They are effective for feature selection, classification tasks in drug discovery, and toxicity prediction (Table 1 and Figure 4) [56].

In a study focusing on aging and the development of pharmaceutical interventions, researchers utilized the ML model to predict the lifespan-extending potential of chemical compounds using data from the DrugAge database. The model, built using the random forest algorithm, achieved a high classification performance with an area under the curve (AUC) score of 0.815. The top features of the model included descriptors related to atom and bond counts and topological and partial charge properties. When applied to an external database, the model categorized compounds with high predictive probabilities for lifespan extension into groups, including flavonoids, fatty acids and conjugates, and organooxygen compounds [57].

Deep Learning

DL refers to a subfield of ML that focuses on developing and applying artificial neural networks (ANNs) with multiple layers. DL algorithms, such as neural networks and CNNs, have gained significant attention in pharmacological applications.

**Figure 5:**
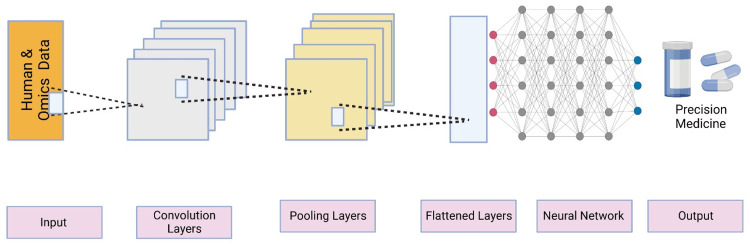
Convolutional neural networks technique in precision medicine. Convolutional neural networks consist of convolutional, pooling, and fully connected layers. The convolutional layer applies filters to extract features from input images, while the pooling layer reduces the dimensionality of the data. The fully connected layer makes predictions based on the extracted features. CNNs automatically learn and extract relevant features, making them effective for image understanding and precision medicine. The image is created by the authors of this study.

Neural networks are composed of interconnected nodes or "neurons" that mimic the structure and function of biological neurons. They are designed to process and learn from complex patterns in data. Neural networks consist of input layers, hidden layers, and output layers. By iteratively adjusting the connections between neurons, neural networks can learn to recognize and predict patterns in the data (Table 1 and Figure 5).

CNNs, a specific type of neural network, are particularly suitable for image analysis tasks. They use a mathematical operation called convolution to process pixel data in images. CNNs are specifically designed to extract hierarchical features from images by applying convolutional filters to capture local patterns and then aggregating them to recognize higher-level features. CNNs have revolutionized image analysis tasks, enabling accurate image classification, segmentation, and object detection.

DL algorithms, including neural networks and CNNs, have shown great potential in various pharmacological applications. Some key applications of DL in pharmacology are as follows: (1) image analysis - DL algorithms have been successfully applied to image analysis tasks in pharmacology. CNNs, in particular, have been utilized for accurately classifying and segmenting medical images, such as histopathological images for cancer diagnosis, radiological images for disease detection, and microscopy images for drug discovery. (2) Molecular structure prediction - DL algorithms have been employed for predicting molecular structures and properties. For example, molecular convolutional neural networks have been used to predict the 3D structure of molecules and to model their properties, aiding in drug discovery and optimization. (3) Chemical property optimization - DL techniques have been applied to optimize the chemical properties of drug compounds. By training neural networks on large databases of chemical compounds and their associated properties, DL models can suggest modifications to existing compounds or propose new compounds with desired properties [58-61].

A study introduced a DL approach using CNNs for optical coherence tomography (OCT) image analysis of pharmaceutical solid dosage forms. The CNNs are applied to in-line and at-line OCT data for monitoring film-coated tablets and pellets. Performance is compared to established algorithms and validated against human annotations and microscopy images. The approach achieves real-time operation, handles image noise and appearance changes, and outperforms existing algorithms. This advancement in real-time analysis of challenging industrial OCT images holds promise for improved pharmaceutical applications [62].

These DL applications have the potential to accelerate drug discovery, improve diagnosis and treatment, and optimize chemical synthesis processes in pharmacology. Despite the promise and success of DL in pharmacology, there are several challenges and limitations that need to be addressed.DL algorithms typically require large amounts of labeled data for training. In pharmacology, obtaining high-quality labeled datasets can be challenging due to the limited availability of labeled samples, especially in specialized areas such as rare diseases. Additionally, ensuring the quality and reliability of data is crucial to prevent biases and inaccuracies in the models.

Supervised, Unsupervised, and Reinforcement Learning Approaches

*Supervised learning:* In supervised learning, models learn from labeled training data to make predictions or classify new instances. In pharmacology, supervised learning is used for drug-target interaction prediction, virtual screening, and toxicity prediction. SVMs and random forests are popular supervised learning algorithms in pharmacological research.

Unsupervised learning: Unsupervised learning aims to discover patterns and structures in unlabeled data. Clustering algorithms, such as k-means and hierarchical clustering, are used to identify similar groups of drugs or patients based on molecular properties or clinical features. Dimensionality reduction techniques, such as Principal Component Analysis (PCA), help visualize and reduce the complexity of high-dimensional datasets.

Reinforcement learning: While less commonly used in pharmacological research, reinforcement learning (RL) has potential applications in personalized medicine and dose optimization. RL agents learn optimal decision-making policies by interacting with the environment and receiving rewards or penalties. RL has been explored for drug dosage determination and adaptive treatment strategies [63].

Feature Selection and Dimensionality Reduction Techniques

Feature selection: This technique is crucial to identifying informative features from high-dimensional datasets in pharmacological research. Methods like Recursive Feature Elimination (RFE) and Least Absolute Shrinkage and Selection Operation (LASSO) regression are used to select relevant descriptors for drug-target interaction prediction or patient stratification.

Dimensionality reduction: These techniques like PCA and t-Distributed Stochastic Neighbor Embedding (t-SNE) help visualize and interpret complex pharmacological datasets by transforming high-dimensional data into lower-dimensional representations while preserving essential information. They aid in better understanding and decision-making [64-70].

Ethical considerations and challenges

Integrating AI in pharmacological research holds immense potential for advancing healthcare outcomes. However, to ensure the responsible and ethical use of AI, researchers and healthcare stakeholders must address the challenges related to data privacy, bias, fairness, adoption, and integration into clinical practice. Some of the issues and probable solutions are highlighted in Table 3 [71-75].

**Table 3:** Addressing ethical concerns in the application of artificial intelligence (AI) in pharmacological research AI has been challenged with several ethical issues that malign its value and raise concerns. Evidence suggests that these issues can be addressed by having inbuilt system checks, robust methodology, and overall transparency.

Problem	Solution
Data privacy and security in AI-driven pharmacological research	Design AI models with privacy by design principles
	Implement strong encryption and access controls
	Adhere to data protection regulations and obtain informed consent from patients
	Ensure data anonymization when necessary
Bias and fairness issues in AI models	Curate and preprocess training data to ensure diversity and minimize biases
	Monitor and audit AI systems to identify and correct biases during deployment
	Employ fairness-aware algorithms and implement fairness constraints to ensure equitable treatment
Integration of AI into clinical practice	Implement comprehensive training programs and educational initiatives for healthcare professionals
	Incorporate AI into existing clinical workflows in a user-friendly manner
	Address technical challenges such as interoperability and standardization of data formats
	Overcome organizational barriers and concerns about job displacement
	Collaborate with policymakers, healthcare institutions, and AI developers to create a supportive environment

Future perspectives

AI has revolutionized pharmacology, with emerging trends including explainable AI, reinforcement learning, and the integration of AI with blockchain and the internet of medical things (IoMT) [76,77].

Explainable AI for Enhanced Transparency and Interpretability

Explainable AI will provide transparency and interpretability, explaining the process behind AI predictions and decisions. Rule-based systems, local interpretable model-agnostic explanations (LIME), and SHAP reveal drug design and response factors, enhancing trust in decision-making.

Reinforcement Learning and Generative Models for Novel Drug Design

RL will optimize drug design by training agents to maximize rewards. It will utilize the understanding of the interaction between drugs and biological systems to generate novel molecules with desired properties. Generative models like GANs and VAEs will accelerate the discovery of potential drug candidates and effective therapies.

Integration of AI With Other Technologies Like Blockchain and IoMT

Integrating blockchain into pharmacological research will ensure the security and integrity of medical data, while IoMT will enable real-time analysis of patient data. This will ensure privacy, data sharing, personalized treatment, and medication adherence.

## Conclusions

AI plays a crucial role in pharmacology, revolutionizing the field and enhancing various aspects of drug discovery, development, research, and clinical practice. AI models, such as ML and NLP, are able to analyze large volumes of data, identify patterns, and make predictions. In drug discovery, AI assists in the identification of potential drug compounds and suitable patient populations, leading to more efficient and targeted therapies. AI also aids in real-world data mining, therapeutic drug monitoring, and optimizing clinical trial design and analysis. By automating processes and improving decision-making, AI enables personalized medicine and increases efficiency in the pharmaceutical industry. AI in pharmacology is a significant step toward improving patient outcomes and advancing healthcare.
